# 1538. Is there value in performing yearly screening for latent tuberculosis infection by interferon-gamma release assay among patients living with HIV in non-endemic settings?

**DOI:** 10.1093/ofid/ofad500.1373

**Published:** 2023-11-27

**Authors:** Carlo Foppiano Palacios, Amit Achhra, Lydia A Barakat, Michael Virata, Richie Hao, John D Baxter, Maricar F Malinis

**Affiliations:** Yale University, New Haven, Connecticut; Yale school of Medicine, New Haven, Connecticut; Yale School of Medicine, New Haven, Connecticut; Yale University, New Haven, Connecticut; Yale University, New Haven, Connecticut; Cooper University Healthcare, Camden, New Jersey; Yale University, New Haven, Connecticut

## Abstract

**Background:**

People with HIV (PWH) are at increased risk of reactivation of tuberculosis (TB) from latent tuberculosis infection (LTBI). Current guidelines recommend all PWH be initially screened for LTBI with either interferon-gamma release assays (IGRA) or tuberculin skin testing (TST). Also, repeat screening is recommended in either PWH with CD4 count < 200 cells/mm^3^ after initial negative screen or any PWH with new TB exposure risk. Despite such guideline, there are centers that continue to screen LTBI in all PWH with annual IGRA testing. Our study evaluated the utility of universal yearly LTBI screening by IGRA among PWH in non-endemic settings.

**Methods:**

A retrospective chart review of PWH in care from 2017-2021 at two urban academic medical centers. Demographics and annual IGRA results were collected. Patients were further stratified into three groups based on IGRA seroconversion: those with negative to positive (Group A), indeterminate to positive (Group B), and negative to indeterminate (Group C). Data for 3 groups, included risk factors for TB, chest imaging results, HIV data, and treatment. Descriptive statistics, chi-square, and Welch’s ANOVA were performed.

**Results:**

A total of 2694 PWH were in care, comprised of 66% male, 50% Black, and 23% LatinX. Of the 2694, 2255 (84%) had a negative baseline IGRA. Seventy-two patients (2.7%) had IGRA seroconversion: 39 in Group A, 1 in Group B, and 32 in Group C (**Table 1**). Group A had lower CD4 counts (p=0.04) and higher HIV viral loads (p=0.03) than Group C.

Only 12 PWH (0.5%) with negative baseline IGRA developed new LTBI, with an incidence 1.1 cases/1000 patient-years. Of these, 8 PWH (67%) had ≥ 1 TB risk factor and none had CD4< 200. Travel to endemic region (33%) was most common risk factor. Among those with new LTBI, N=6/7 had confirmation of LTBI with repeat positive IGRA after initial seroconversion. Nine (75%) patients completed LTBI treatment and none developed tuberculosis.

Features of patients with conversion to a positive or indeterminate QuantiFERON

**Figure:**
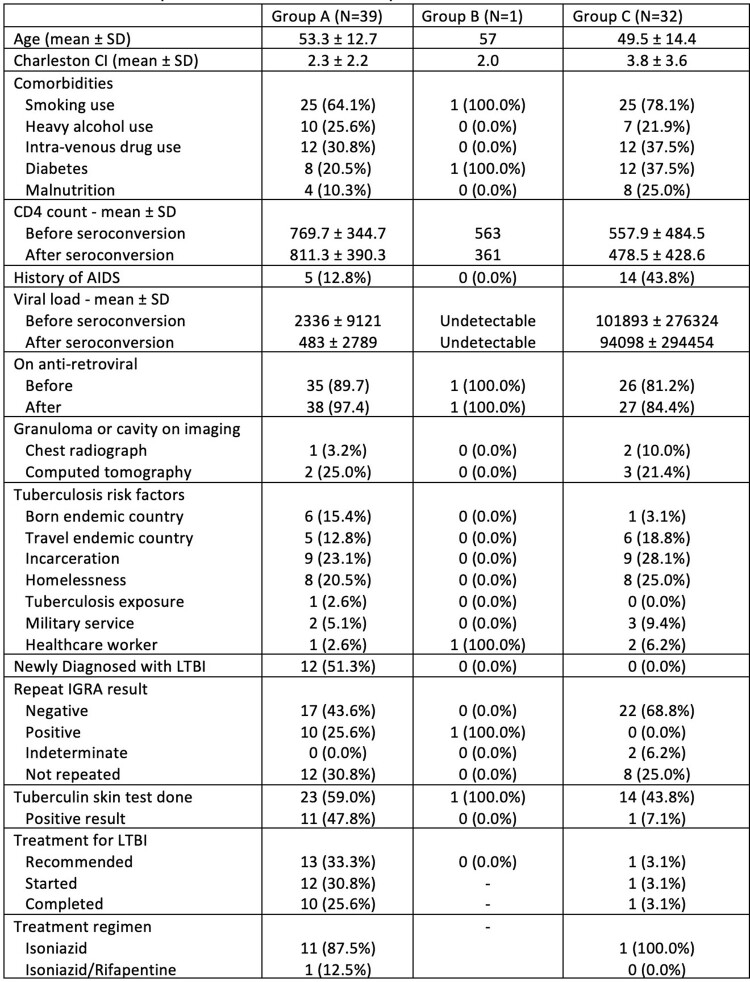

**Conclusion:**

In two large diverse clinics in non-TB endemic settings, the true incidence of developing LTBI in PWH was rare among those with negative baseline IGRA. Due to low yield of cases with universal annual screening, our findings support the targeted approach recommended by current guidelines.

**Disclosures:**

**Michael Virata, MD**, Gilead Sciences: Advisor/Consultant|Janssen: Advisor/Consultant|ViiV Healthcare: Advisor/Consultant

